# Characterizing the admixed African ancestry of African Americans

**DOI:** 10.1186/gb-2009-10-12-r141

**Published:** 2009-12-22

**Authors:** Fouad Zakharia, Analabha Basu, Devin Absher, Themistocles L Assimes, Alan S Go, Mark A Hlatky, Carlos Iribarren, Joshua W Knowles, Jun Li, Balasubramanian Narasimhan, Steven Sidney, Audrey Southwick, Richard M Myers, Thomas Quertermous, Neil Risch, Hua Tang

**Affiliations:** 1 Department of Genetics, Stanford University School of Medicine, 300 Pasteur Drive, Stanford, CA 94305, USA; 2 Institute for Human Genetics, University of California, San Francisco, 513 Parnassus Ave., San Francisco, CA 94143, USA; 3 HudsonAlpha Institute for Biotechnology, 601 Genome Way, Huntsville, AL 35806, USA; 4 Division of Cardiovascular Medicine, Stanford University School of Medicine, 300 Pasteur Drive, Stanford, CA 94305, USA; 5 Division of Research, Kaiser Permanente, 2000 Broadway, Oakland, CA 94612, USA; 6 Department of Health, Research and Policy, Stanford University School of Medicine, 150 Governors Lane, Stanford, CA 94305, USA; 7 Department of Human Genetics, University of Michigan, 1241 E. Catherine St., Ann Arbor, MI 48109, USA; 8 Department of Infectious Diseases, Stanford University School of Medicine, 300 Pasteur Drive, Stanford, CA 94305, USA; 9 Department of Epidemiology and Biostatistics, University of California, San Francisco, 185 Berry Street, San Francisco, CA 94107, USA

## Abstract

**Background:**

Accurate, high-throughput genotyping allows the fine characterization of genetic ancestry. Here we applied recently developed statistical and computational techniques to the question of African ancestry in African Americans by using data on more than 450,000 single-nucleotide polymorphisms (SNPs) genotyped in 94 Africans of diverse geographic origins included in the HGDP, as well as 136 African Americans and 38 European Americans participating in the Atherosclerotic Disease Vascular Function and Genetic Epidemiology (ADVANCE) study. To focus on African ancestry, we reduced the data to include only those genotypes in each African American determined statistically to be African in origin.

**Results:**

From cluster analysis, we found that all the African Americans are admixed in their African components of ancestry, with the majority contributions being from West and West-Central Africa, and only modest variation in these African-ancestry proportions among individuals. Furthermore, by principal components analysis, we found little evidence of genetic structure within the African component of ancestry in African Americans.

**Conclusions:**

These results are consistent with historic mating patterns among African Americans that are largely uncorrelated to African ancestral origins, and they cast doubt on the general utility of mtDNA or Y-chromosome markers alone to delineate the full African ancestry of African Americans. Our results also indicate that the genetic architecture of African Americans is distinct from that of Africans, and that the greatest source of potential genetic stratification bias in case-control studies of African Americans derives from the proportion of European ancestry.

## Abstract 2

Genome-wide SNP analyses reveal the admixed African genetic ancestry of African Americans.

## Background

Numerous studies have estimated the rate of European admixture in African Americans; these studies have documented average admixture rates in the range of 10% to 20%, with some regional variation, but also with substantial variation among individuals [1]. For example, the largest study of African Americans to date, based on autosomal short tandem repeat (STR) markers, found an average of 14% European ancestry with a standard deviation of approximately 10%, and a range of near 0 to 65% [1], whereas another study based on ancestry informative markers (AIMs) found an average of 17.7% European ancestry with a standard deviation of 15.0% [2]. By using nine AIMs, Parra and colleagues [3] found substantial variation of European ancestry proportions in African-American populations across the United States, ranging from just over 10% in a Philadelphia group to more than 20% in a New Orleans population. Similar levels (11% to 15%) of European ancestry also were reported by Tishkoff and co-workers [4], based on more than 1,000 nuclear microsatellite and insertion/deletion markers.

Although much attention has been paid in the genetics literature to the continental admixture underlying the genetic makeup of African Americans, less attention has been paid to the within-continental contribution to African Americans, in particular from the continent of Africa. Studies have focused primarily on the matrilineally inherited mitochondrial DNA (mtDNA) and patrilineally inherited Y chromosome [5-7]. These two DNA sources have gained wide prominence owing, in part, to their use by ancestry-testing companies to identify the regional and ethnic origins of their subscribers. Yet these two sources provide a very narrow perspective in delineating only two of possibly thousands of ancestral lineages in an individual.

The majority of African Americans derive their African ancestry from the approximately 500,000 to 650,000 Africans that were forcibly brought to British North America as slaves during the Middle Passage [8,9]. These individuals were deported primarily from various geographic regions of Western Africa, ranging from Senegal to Nigeria to Angola. Thus, it has been estimated that the majority of African Americans derive ancestry from these geographic regions, although more central and eastern locations also have contributed [10-12]. Recent studies of African and African-American mtDNA haplotypes and autosomal microsatellite markers also confirmed a broad range of Western Africa as the likely roots of most African Americans [4,13].

The recent development of high-density single-nucleotide polymorphism (SNP) genotyping assays, used primarily in genome-wide association (GWA) studies, has also provided unprecedented opportunities to address questions related to the evolution and migration patterns of humans. Most of the GWA studies to date have focused on European or European-derived populations of U.S. Caucasians, but a few have included minorities. The latter studies provide unique opportunities to address questions of ancestral origins in admixed populations, such as African Americans and Latinos [14-16].

Although the application of high-density genotyping to a broad range of worldwide indigenous populations has not yet been accomplished, an important first step has been achieved through the recent genotyping of the Human Genome Diversity Panel (HGDP). This effort resulted in nearly 1,000 subjects from 51 populations being genotyped at more than 500,000 polymorphic sites [17,18]. These data now provide a basis for finer-scale analysis of the ancestral origins of admixed groups, such as African Americans and Latinos, in addition to enabling the accurate characterization of genetic and evolutionary relationships among these populations.

In this study, we characterize the African origins of African Americans by making use of the high-density genotype data generated for 94 HGDP indigenous Africans from differing geographic and linguistic groups, including 21 Mandenka from West Africa, 21 Yoruba from West Central Africa, 15 Bantu speakers from Southwestern and Eastern Africa, 20 Biaka Pygmy and 12 Mbuti Pygmy from Central Africa, and five San from Southern Africa [18]. These subjects are used to represent the potential African ancestors of 136 African Americans recently genotyped in a GWA study of early-onset coronary artery disease (ADVANCE) [19]. In addition, we include 38 U.S. Caucasian subjects from ADVANCE to represent the European ancestors of the African Americans.

The use of high-density SNP data for ancestral reconstruction presents some unique statistical and computational challenges. To this end, we previously developed analytic techniques for estimating individual ancestry (IA) from multiple populations (*frappe*), as well as for the reconstruction of ancestry blocks in admixed individuals (*saber*) by using data from more than 450,000 SNP genotypes [20,21]. Here, we provide a unique application of *saber *to identify the ancestral origins of each of the more than 450,000 genotypes in African-American individuals, to reduce the analysis to those genotypes that are exclusively of African origin. We note that 58 of the ADVANCE African Americans were also participants of the CARDIA study and had previously been analyzed with 42 Ancestry Informative Markers [22]. We also used principal components analysis (PCA) to define the genetic structure, and in particular the African genetic structure, underlying African Americans. Another recent study used principal components analysis for the African populations of HGDP, but did not relate those results to African Americans [23]. To our knowledge, the analyses reported here represent the first effort to characterize the African origin of African Americans by isolating the African-derived genome in each African American individual.

## Results

### African and European ancestry in African Americans

**Figure 1:**
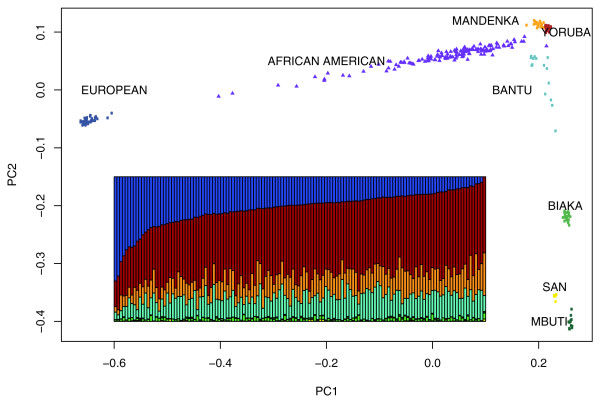
Principal components analysis of Africans, U.S. Caucasians, and African Americans. Inset bar plot displays individual ancestry estimates for African Americans from a supervised structure analysis by using *frappe *with K = 7, fixing six African and one U.S. Caucasian populations. The color scheme of the bar plot matches that in the PCA plot.

Principal components analysis of more than 450,000 SNPs, including all populations (Africans, African Americans, and US Caucasians), revealed, as expected, a major separation between the African and U.S. Caucasian populations along the first principal component (PC1), whereas the second principal component (PC2) led to the separation of the various African groups (Figure 1). The two pygmy populations (Biaka, Mbuti) and the San of South Africa are well separated from the other African groups, whereas a greater genetic affinity appears to exist between the Mandenka of West Africa, the Yoruba of Central West Africa, and the Bantu speakers, who derive from Kenya and Southwestern Africa. It is also clear in Figure 1 that the African Americans lie on a direct line between the European Americans and the West Africans, reflecting their varying levels of admixture between these two ancestral groups.

These results were confirmed in the estimation of IA by using the program *frappe *(also in Figure 1). The amount of European ancestry shows considerable variation, with an average (± SD) of 21.9% ± 12.2%, and a range of 0 to 72% (Table 1). The largest African ancestral contribution comes from the Yoruba, with an average of 47.1% ± 8.7% (range, 18% to 64%), followed by the Bantu at 14.8% ± 5.0% (range, 3% to 28%) and Mandenka at 13.8% ± 4.5% (range, 3% to 29%). The contributions from the other three African groups were quite modest, with an average of 1.7% from the Biaka, 0.5% from the Mbuti, and 0.3% from the San. In the bar plot of *frappe *estimates, individuals (vertical bars) are arranged in order (left to right) corresponding to their value on the first PC coordinate. Clearly, this order correlates nearly perfectly with a decreasing proportion of European ancestry (Figure S1 in Additional file 1). Thus, the most important source of genetic structure in African Americans is based on the degree of European admixture.

**Table 1:** Estimates of European ancestry and proportional African ancestries in African Americans by US region of birth

U.S. region of birth	Number^a^	European ancestry (%)	Total African ancestry (%)^b^					
			**Mandenka**	**Yoruba**	**Bantu**	**Biaka**	**Mbuti**	**San**
			
West	58 (58)	19.9 ± 8.5	18.9 ± 4.1	64.0 ± 5.3	13.7 ± 4.3	1.1 ± 0.8	0.2 ± 0.2	2.0 ± 0.5
South	12 (10)	24.0 ± 15.6	22.6 ± 5.7	60.0 ± 9.5	14.2 ± 5.4	1.1 ± 0.7	0.2 ± 0.4	1.9 ± 1.0
Midwest	4 (4)	19.4 ± 10.2	19.4 ± 2.0	64.0 ± 5.5	13.1 ± 5.5	0.9 ± 0.9	0.3 ± 0.3	2.2 ± 0.7
Southwest	2 (2)	17.0 ± 6.5	21.4 ± 0.7	65.1 ± 1.0	10.5 ± 0.3	1.1 ± 0.4	0.1 ± 0.0	1.7 ± 1.0
All	136 (128)	21.9 ± 12.2	19.2 ± 4.0	63.7 ± 4.9	13.8 ± 3.8	1.0 ± 0.8	0.2 ± 0.3	2.0 ± 0.6

^a^Numbers in parentheses are those used for estimation of African ancestries after removal of eight individuals with high values of European ancestry; birth-location information was missing for 60 individuals.

^b^Based on *frappe *analysis of African genotypes only (n = 128).

### African components of ancestry in African Americans

We estimate that, on average, nearly 80% of the ancestry in our samples of African Americans is of African origin. A careful examination of the African component of ancestry in the African Americans is facilitated by restricting the analysis to those portions of their genomes that are exclusively of African origin. To do so, we used the program *saber *to infer European- *versus *African-derived alleles for each individual, and retained for analysis only those loci that had a high probability of harboring two African-derived alleles, while denoting the other genotypes as missing. For these and all subsequent analyses, we included the 128 African Americans whose estimated African ancestry exceeded 55%, based on the initial *frappe *analysis (see Methods).

**Figure 2:**
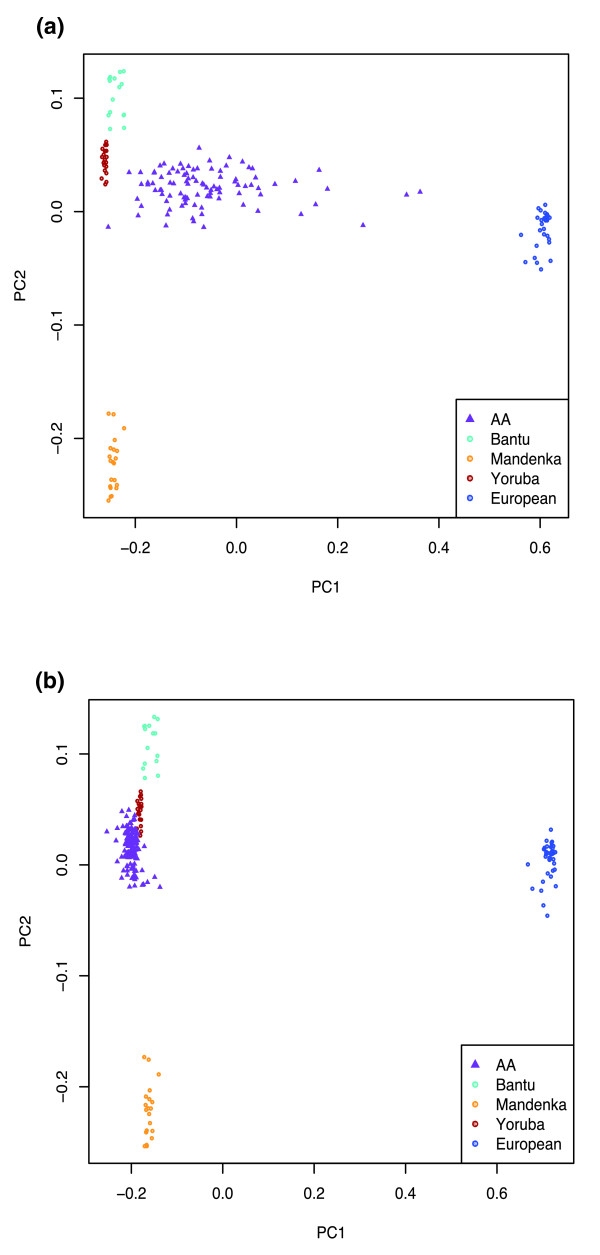
Principal components analysis of Africans, U.S. Caucasians, and African Americans including **(a)** all genotypes, and **(b)** only the genotypes of African origin in the African Americans. Comparison of (a) and (b) demonstrates the effective elimination of the European ancestry component from African Americans by using *saber*.

As a validation of the accuracy of this partitioning procedure, we performed PCA on the combined set of U.S. Caucasians, Africans, and the African Americans with putative non-African-derived genotypes removed (that is, coded as missing). For comparison, we also examined the results of the same analysis, but including all of the genotype data of the African Americans. For these analyses, we included only the three African population groups that, based on the first analysis, contributed significantly to the African Americans (the Mandenka, Yoruba, and Bantu). As shown previously, when all genotypes are included, the African Americans lie intermediate between the Africans and European Americans, at varying distances based on their degree of admixture (Figure 2a). By contrast, when only the putative African-derived genotypes in the African Americans are included, the African Americans now cluster tightly with the Africans (Figure 2b). This result provides confidence that the application of *saber *has enabled us to partition accurately the genomes of the African Americans with regard to European *versus *African ancestry.

We then characterized the African ancestry in African Americans by performing PCA and estimating IA with *frappe *by using the U.S. Caucasians, Africans, and African Americans, with non-African genotypes removed. To determine whether we could distinguish the African populations from one another, we first ran *frappe *including all the 94 African individuals (setting K = 6). This unsupervised analysis unambiguously separated the San and Pygmy populations from the West Africans and, to a lesser degree, the three West African populations (Yoruba, Mandenka, and Bantu). To be confident in the groupings of the West African population, we performed a series of leave-one-out *frappe *analyses that include 57 individuals from the three West African populations: in each *frappe *run, we fixed all individual within their respective populations except for one, whose ancestry was allowed to be admixed and estimated (see Methods). Results are given in Figure S2 in Additional file 1. The close genetic relationship of these three groups is evidenced by the imperfect ancestry allocation to an individual's own population. However, in every case, *frappe *assigns the majority ancestry to an individual's own population, and in most cases, the large majority. The Bantu appear to have closest ancestry to the Yoruba. This is consistent with the Nigerian origins of the Yoruba and the presumed origins of the Bantu from the southwestern modern boundary of Nigeria and Cameroon [24], and the subsequent migration of the Bantu east and south [5,25].

**Figure 3:**
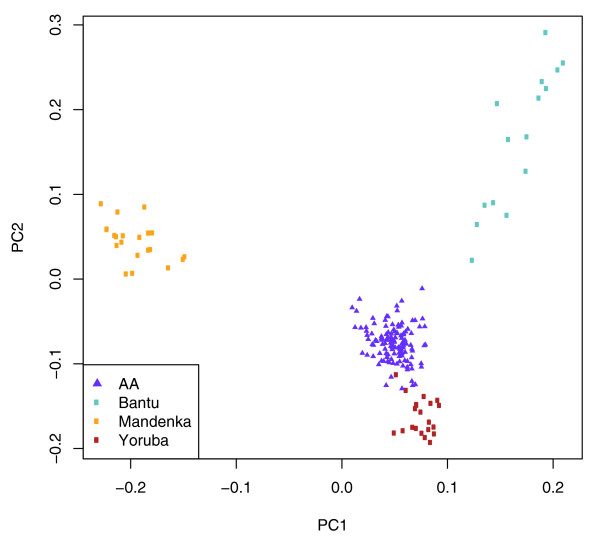
Principal components analysis of three West and Central West African populations (Mandenka, Yoruba, and Bantu) and African Americans by using only African-origin genotypes in the African Americans.

Figure 3 displays the PCA results of the African Americans and the three closely related African populations (Yoruba, Mandenka, and Bantu). Several features are worth comment. First, despite their genetic similarity, PCA shows clear separation among the Yoruba, Mandenka, and Bantu populations, based on the first two PCs. Second, Figure 3 reveals that the African Americans are placed as a single cluster in the convex hull defined by the three African groups.

**Figure 4:**
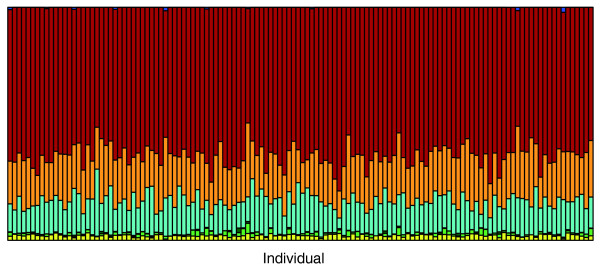
Individual ancestry estimates in African Americans by using only their African genotypes, from a supervised structure analysis with *frappe*, including all six African populations and U.S. Caucasians as fixed (K = 7). Color coding of populations is the same as that in Figure 1.

Figure 4 presents the results of the *frappe *analysis of the 128 African Americans, in which the six HGDP African populations and Caucasians from ADVANCE were included in the analysis as fixed groups, and proportional ancestry estimated for the African Americans. Consistent with Figure 1, Figure 4 shows that all African Americans are estimated to have significant ancestry from each of the three West and Central West African groups (Mandenka, Yoruba, and Bantu), with only modest variation among individuals in their ancestral proportions from these three groups. As expected, little to no European ancestry is estimated in this *frappe *analysis.

Table 1 provides the averages and standard deviations of IA derived from the *frappe *analysis described earlier (Figure 4) for the African components of African ancestry for the 128 African Americans. Overall, we estimate within-Africa contributions of 64%, 19%, and 14% from Yoruba, Mandenka, and Bantu, respectively. The variances for the various African IA components are much smaller than those for the European IA and are roughly similar across groups (SD ranging from 0.038 to 0.049). These observations are consistent with visual inspection of the bar chart in Figure 4, that African Americans generally derive substantial ancestry from all three West and Central West African population groups. We also note from Table 1 that no significant differences exist among African-American subgroups defined by U.S. region of birth, in terms of IA estimates for any African ancestral component, nor are any significant differences in IA found, based on gender (data not shown).

Thus, the PC and *frappe *analyses of the 128 African Americans based only on their African-derived genotypes suggest a lack of genetic structure within the African component of their ancestry. To assess this question further, we performed an additional PC analysis on only the African Americans, including only the African-derived genotypes for each individual.

**Figure 5:**
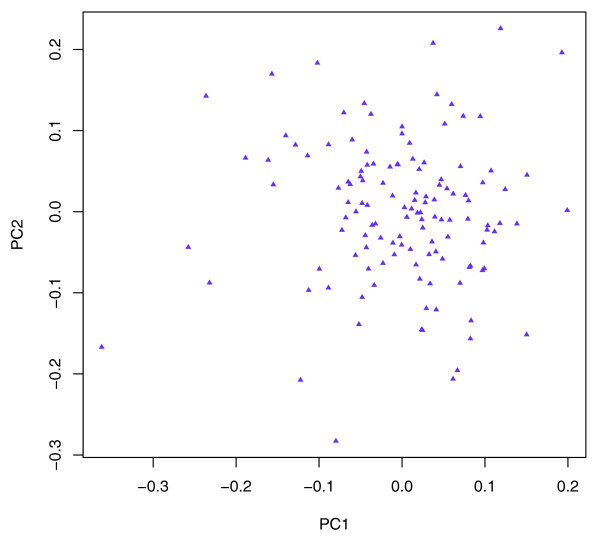
Principal components analysis of African Americans based on African-derived genotypes only. Little evidence for structure exists in the African component of ancestry.

Figure 5 shows the PCA restricted to African-derived genotypes within the African Americans. In this case, each PC accounts for a very modest amount of variance, and no clear pattern is evident. The distribution of the proportion of variance explained by each PC revealed a continuous distribution with no outliers (data not shown).

To confirm that this lack of structure was not an artifact of removing genotype data, we performed a similar PC analysis on the original 94 Africans, but randomly deleting genotypes from these subjects at a rate comparable to the genotype removal rate in the African Americans (see Methods). Results are shown in Figure S3a (full genotype data) and Figure S3b (genotype data removed) in Additional file 1. As can be seen, the two figures appear nearly identical, each demonstrating the structure that exists among these African populations. Thus, the deletion of genotypes did little to diminish the display of population structure, and so the lack of structure that we observed within the African Americans (after removing non-African genotypes) is unlikely due to missing genotype data.

Another question relates to potential impact of missing genotypes on the *frappe *analysis of the African Americans. Individuals with high levels of European ancestry (who have more genotype data removed) provide less information regarding their African ancestral components, and thus the variance of the African components of IA increases with the amount of European ancestry, but not in a directional way.

## Discussion

As expected, PCA on our entire sample revealed the greatest genetic differentiation between the US Caucasians and the Africans, with the African Americans intermediate between them, reflecting their recent admixture between ancestors from Europe and Africa. Our estimate of European individual admixture (IA) in the African Americans was also roughly consistent with prior studies [3], with an average of 21.9%. We found considerable variation among individuals in terms of European IA, and a number of individuals with particularly high European IA values (eight individuals of 136, or 6% with values greater than 45%).

Prior studies focusing on mtDNA and Y chromosomes have found a greater African and lesser European representation of mtDNA haplotypes compared with Y chromosome haplotypes in African Americans, suggesting a greater contribution of African matrilineal descent compared with patrilineal descent [6,7]. For example, Kayser and colleagues [6] estimated that 27.5% to 33.6% of Y chromosomes in African Americans are of European origin, compared with 9.0% to 15.4% of mtDNA haplotypes.

One study of nine short tandem repeat (STR) loci compared the Y chromosomes of African Americans with those of various African populations, including West Africans, West Central Africans (Cameroon), South Africans, Mbuti Pygmies, Mali, San, and Ethiopians [6]. In a multiple dimensional scaling analysis, these authors placed the African Americans in the middle of these African groups, suggesting origins from multiple African populations. However, they also found that they could not differentiate the Y-chromosome distributions of West African and West Central African groups, presumably a major source of ancestry for African Americans.

Another study of mtDNA haplotypes in African Americans and different African populations found that more than 50% of the African-American mtDNAs exactly matched common haplotypes shared among multiple African ethnic groups, whereas 40% matched no sequences in the African database they referenced [26]. Fewer than 10% of African-American mtDNA haplotypes matched exactly to a single African ethnic group. The haplotypes that did match were more often found in ethnic groups of West African or Central West African than of East or South African origin.

The most extensive examination of mtDNA haplotypes in Africans and African Americans [13] used mtDNA data from a large number of African ethnic groups spread around the continent. These authors observed large similarities in mtDNA profiles among ethnic groups from West, Central West, and South West Africa, with a continuous geographic gradient. As observed previously [26], these authors also found that many mtDNA haplotypes were widely distributed across Africa, making it impossible to trace African ancestry to a particular region or group, based on mtDNA data alone. These authors also estimated the proportionate ancestry within Africa based on African American mtDNA haplotypes as 60% from West Africa, 9% from Central West Africa, 30% from South West Africa, and minimal ancestry from North, East, Southeast, or South Africa.

These studies all suggest close genetic kinship among various West African, Central West African, and South West African ethnic groups. A prior analysis of genetic structure among the African populations included in the HGDP based on 377 autosomal STR loci was able to define distinct genetic clusters for the Biaka, Mbuti, and San; however, the study lacked the power to differentiate the Mandenka, Yoruba, and Bantu groups [27]. Similarly, another study examining two ethnic groups from Ghana (Akan and Gaa-Adangbe) and two from Nigeria (Yoruba, Igbo), based on 372 autosomal microsatellite markers in 493 individuals, did not differentiate these groups by genetic cluster analysis and found only modest genetic differences between them [28]. In contrast, greater resolution of African ethnic groups, particularly for the Mandenka and Yoruba, was possible in our analysis, based on more than 450,000 SNPs. We note that, in a recent study of malaria, PCA distinguished the HapMap YRI individuals from the Mandenka individuals in the Gambian sample on the basis of 100,715 SNPs; however, admixture analysis with a few selected markers did not reveal clear clusters that correspond to self-reported ancestry [29].

It is of interest to compare our African admixture estimates to descriptions of proportional representation of various African groups to the Middle Passage and slave trade occurring in post-Columbian America. A highly detailed census based on historic records has been documented by several authors [10-12]. Africans were deported from numerous locations along the broad western coast of Africa, ranging from Senegal in the far west all the way down to Angola in the southwest. In addition, a smaller number of slaves were taken from the southeast of Africa. In terms of numbers, the largest group, approximately 50% to 60%, derived from Central and Southern West Africa and the Bight of Biafra; approximately 10% from Western Africa; 25% to 35% from the West Coast in between (Windward Coast, Gold Coast, and Bight of Benin), and the remaining 5% from Southeast Africa [7]. These estimates show considerable consistency with our results, which also indicated the largest ancestral component of African Americans to be from Central West Africa, followed by West Africa and Southwest Africa. However, because we did not have groups representative of Southeastern and other parts of Southern Africa, we may have underestimated their ancestral representation among African Americans.

It is important to note that considerable migration has occurred among African ethnic groups over the past three millennia or more. For example, the two Bantu groups included in our analysis originated from a more-central African location (Nigeria-Cameroon) several millennia ago, making precise geographic localization of African ancestry difficult [30]. This difficulty is also reflected in the close genetic relationships among the various West, West Central, and South West African groups, who also show considerable overlap in terms of mtDNA haplotypes.

Our results are based on examination of the entire autosomal genome and, therefore, provide a more-robust picture of the admixed African ancestry of individual African Americans compared with prior analyses, which focused on only a single locus (mtDNA or Y chromosome). We found all African Americans in our sample to be admixed, with representation from various geographic regions of Western Africa. The amount of variation in the African components of ancestry among the African Americans was quite modest, suggesting considerable similarity in African genetic profiles among African Americans. Thus, African ancestry testing based on a single locus, such as the mtDNA or Y chromosome, as is commonly done by ancestry-testing companies, provides only a very limited, and in many cases, misleading picture of an individual's African ancestry [31].

An important limitation in our analysis is the modest number of African subjects and groups represented. However, we were clearly able to exclude certain African ethnic groups as contributing substantially to African Americans, such as the two Pygmy and San groups. Furthermore, the close genetic similarity observed among West, Central West, and Southwest African ethnic groups (such as the Mandenka, Yoruba, and Bantu), found by us and others [28], suggests that precise identification of ancestry for African Americans may be difficult, even with the inclusion of additional ethnic groups.

Very recently, the limited range of African groups included in population genetic studies of Africans was addressed in a landmark study of 113 geographically diverse African ethnic groups by Tishkoff and co-workers [4]. These authors included 848 microsatellite, 476 indel, and four SNP markers. to examine genetic structure among these groups, as well as among 98 African Americans from four U.S. recruitment sites. In a genetic cluster analysis, they found only modest differentiation among West Africans, similar to the findings from other studies of a subset of these groups, based on a comparable number of markers. They also estimated proportionate African ancestry among their African Americans in a structured analysis including African ethnic subgroups, allowing the African Americans to be admixed. Comparable to our results, within the African Americans, they also found the majority African ancestry to be West, Central West, and Southwest African, including Bantu and non-Bantu speakers, with somewhat greater representation of the Bantu speakers (about 50% of the African total component) than the Western non-Bantu speakers (for example, Mandenka, about 30% of the African total component). Larger collections of indigenous African populations, such as those described earlier [4], when assayed with dense genotyping arrays, as done in this study (to allow finer genetic differentiation), will likely add further clarification of the African ancestral origins of African Americans.

The results of our analysis also strongly point to random mating among African Americans with respect to the African components of their ancestry. This is reflected both by the modest variances we observed in the African IA components, and also by the lack of structure in the PC analysis of African Americans with non-African genotypes removed. This conclusion is consistent with the idea that, for most African Americans, specific African origins are mixed or unknown or both and do not affect social characteristics that influence the choice of mate. It is also consistent with the notion that the African slaves brought to North America were mixed with regard to their geographic and ethnic ancestry and language [32]. By contrast, considerably greater variation in the proportion of European ancestry was found within the African Americans in our study. This high level of variation in European ancestry may reflect recent admixture or nonrandom mating (for example, as seen in Latino populations [33]), or both; these questions require additional study.

## Conclusions

African Americans typically have African and European genetic ancestry. We sought to characterize the African ancestry of African Americans by using data on more than 450,000 SNPs genotyped in 94 Africans of diverse geographic origins, as well as 136 African Americans and 38 U.S. Caucasians. To focus on African ancestry, we reduced the data to include only those genotypes in each African American that are African in origin. We found that all the African Americans are admixed in the African component of their ancestry, with estimated contributions of 19% West (for example, Mandenka), 63% West Central (for example, Yoruba), and 14% South West Central or Eastern (for example, Bantu speakers), with little variation among individuals. Furthermore, we found little evidence of genetic structure within the African component of ancestry in African Americans, but significant structure related to the proportion of European ancestry. These results are consistent with mating patterns among African Americans that are unrelated to African ancestral origins, cast doubt on the general utility of mtDNA or Y-chromosome markers alone to delineate the full African ancestry of African Americans, and show that the proportion of European ancestry is the leading source of stratification bias in genetic case-control studies of African Americans.

## Materials and methods

### Selection of populations and individuals

Individuals included in analyses presented here come from two studies. A total of 102 indigenous African individuals and their genotype data were obtained from the Human Genome Diversity Project (HGDP) and comprised five San, 22 Biaka Pygmy, 13 Mbuti Pygmy, 22 Mandenka, 21 Yoruba, 11 Kenyan Bantu, and eight Southwest African Bantu (one Pedi, one Southern Sotho, two Tswana, one Zulu, two Herero, and one Ovambo). In total, eight individuals were removed from analyses for the following reasons: three Kenyan Bantu had significant Middle Eastern ancestry, based on previous analysis [18]; and three additional Kenyan Bantu and two Mandenka were removed because they were first cousins to other included subjects. This left a total of 94 indigenous Africans for analysis. The 136 self-described African-American individuals studied represent a subset of participants of the Atherosclerosis, Vascular Function and Genetic Epidemiology (ADVANCE) study [19] selected for genotyping in the context of a GWA case-control study of early-onset coronary artery disease (CAD). From the ADVANCE study, we also randomly sampled 38 of 590 US Caucasians to anchor the European component of African-American ancestry. Thus, in total, 268 individuals are included in this study.

All ADVANCE subjects were recruited from the membership of Kaiser Permanente of Northern California. Among the 136 African Americans, 49 (36%) were affected with CAD (with first presentation at younger than 45 year for male and 55 years for female subjects), and 36 (26.4%) were male subjects. Of the 87 controls, frequency matched by age to the cases, 58 represented participants in the Coronary Artery Risk Development in Young Adults (CARDIA) study originally recruited at the Kaiser Oakland field center who attended the study's Year 15 examination in 2000 to 2001 [19,34]. For 76 (55.9%) of these African-American individuals, we had information on state of birth, with 58 stating they were born in the West (California), 12 in the South (Alabama, Louisiana, Mississippi, Virginia), four in the Midwest (Indiana, Michigan, Missouri, Ohio), and two in the Southwest (Texas). The description of recruitment of these subjects can be found elsewhere [35].

### Genotyping and marker selection

Genotype data were derived from two different research projects. The HGDP individuals were genotyped on the Illumina 650 K Beadarray; experimental protocol and SNP quality-control analysis for the HGDP project and genotyping results were described previously [18,36]. In total, 938 individuals and 642,690 autosomal SNPs passed all quality-control criteria. Genotype data for U.S. African American and Caucasian individuals were obtained from the ADVANCE study, in which genotyping was performed on the Illumina 550 K Beadarray by the same group of investigators, followed by identical quality-control analysis. After removing markers that were absent from either the HGDP dataset or the ADVANCE dataset, the final combined genotype dataset for all analyses in this study consisted of 454,132 autosomal SNPs.

### Population structure and ancestry estimation

We performed PCAs according to the algorithm described by [36]. Genome-wide European admixture proportions in African-American individuals were estimated by using the program *frappe*, which implements an Estimation-Maximization (EM) algorithm for simultaneously inferring each individual's ancestry proportion and allele frequencies in the ancestral populations [21]. In this analysis, ancestry of the African Americans is allowed to have come from any of the K = 7 ancestral populations: San, Biaka Pygmy, Mbuti Pygmy, Mandenka, Yoruba, Bantu, or European. Ancestries of the indigenous African individuals and U.S. Caucasians were assumed to be homogeneous and fixed. However, to determine the robustness of these assignments for the closely related West and Central West African populations, we performed an additional *frappe *analysis on just these groups (Mandenka, Yoruba, Bantu; n = 57). We fixed all individuals in their respective population groups (Mandenka, Yoruba, or Bantu), except for one, who was allowed to be admixed, and the admixture was estimated. This procedure was repeated 57 times for each individual, so that each person's potential admixture was estimated. In this way, we tested the robustness of the population definitions. If the populations are not distinct, then the individual admixture estimates should appear random; by contrast, if an individual's ancestry is assigned primarily to his or her population of origin, population distinctiveness can be assumed. Furthermore, this analysis provides a closely matched contrast to the African Americans, whose proportionate individual ancestry is estimated in a similar fashion.

### Defining African SNP genotypes

To focus exclusively on the African ancestral component, we removed genotypes containing European-derived alleles from the African-American individuals by using the program *saber*. This program allowed us to infer European *versus *African ancestry for each SNP genotype in an individual [20]. *Saber *implements a Markov-Hidden Markov Model, which infers locus-specific ancestry based on ancestral allele frequencies at each marker, as well as the ancestral haplotype frequencies between pairs of neighboring markers and assumes a block structure for ancestry along a chromosome. For this analysis, *saber *required the genome-wide average European ancestry for each admixed individual, which was estimated by using *frappe*, as described earlier (K = 7). We also supplied the estimated African and European ancestral allele frequencies for all SNPs to *saber*, which improved the estimation of the ancestral haplotype frequencies. *Saber *produces a posterior estimate of European ancestry at each SNP, which concentrates near 0, 0.5 and 1, corresponding to 0, 1, or 2 European-derived alleles. Although it is feasible to infer phase and ancestry jointly by using *saber*, we chose to remove SNP genotypes (as opposed to single alleles) in which at least one allele is European derived. Thus, for a given individual, we were left only with SNP genotypes that were highly likely to be homozygous in African origin. The proportion of genotypes removed for an individual is approximately 1 - α^2^, where α represents the genome-wide estimate of African ancestry for that individual. As a result, the amount of genotype data varied among individuals based on the degree of European *versus *African ancestry. To allow adequate information about the African component of their genome, we excluded eight individuals with estimated European ancestry of 45% or greater, leaving a total sample of 128 individuals with at least 30% of their genotype data retained. The proportion of genotypes retained ranged from 31% to 99%, with a median of 67% and mean of 66%. In terms of proportion of genotypes retained at individual loci, the mean is the same as stated earlier (66%), with a standard deviation of 0.05. Thus, assuming a normal distribution, 95% of the proportions of genotypes retained across loci lie between 56% and 77%. We note that even after removing genotypes, a large number of marker genotypes are retained for each individual, with a minimum of 143,025.

### Genetic structure of the African-derived genome

This analysis focused on IA estimation and PCA based on African-origin SNP genotypes. For IA estimation, we used the program *frappe *with K = 7 (Yoruba, Mandenka, Bantu, Biaka Pygmy, Mbuti Pygmy, San, and U.S. Caucasians as ancestral individuals). U.S. Caucasians were included in the model to ensure that the European ancestral component had been properly removed from all individuals.

In performing PCA of the Africans and African Americans together, our goal was to understand the relationship *between *African Americans and Africans. We focused on the 57 West and Central West Africans in this analysis (Yoruba, Mandenka, and Bantu) because these were the only African populations contributing to African-American ancestry. In this case, a standard PCA would be influenced by the much larger sample size of African Americans compared with any of the African groups. Because we were interested in the projection of the African component of ancestry of the African Americans onto the African structure, we instead performed the PCA 128 times, each time including a different single African American whose non-African genotypes had been removed.

In PCAs involving U.S. Caucasian subjects, the same 38 ADVANCE Caucasians were used. All PCAs were performed by using the statistical package R.

To address the question of whether removal of a varying amount of genotype data among individuals would bias the PC analysis, we performed a genotype-reduction procedure on the 94 indigenous African populations, to mimic the reduction of genotype data among the African Americans. We then performed two PCAs, the first based on complete genotype information, and then another based on the reduced genotype data. Significant differences between the results of these analyses would indicate that some bias occurs simply because of the uneven data reduction; lack of differences would indicate the opposite.

## Abbreviations

ADVANCE: Atherosclerotic Disease Vascular Function and Genetic Epidemiology; AIM: ancestry informative marker; CAD: coronary artery disease; CARDIA: Coronary Artery Risk Development in Young Adults; EM: estimation-maximization; GWA: genome-wide association; HGDP: Human Genome Diversity Panel; IA: individual ancestry; PC: principal component; PCA: principal component analysis; SNP: single nucleotide polymorphism; STR: short tandem repeat.

## Authors' contributions

FZ, HT, and NR conceived of the study, performed the statistical analyses, and drafted the manuscript. AB, DA, and BN contributed to the data analyses. TQ, TLA, JWK, CI, ASG, MAH, and SS are ADVANCE investigators and had the overall responsibility for study design and implementation, including subject recruitment and assessment. RRM, DA, JL, and AS generated high-density SNP genotype data on ADVANCE. All authors contributed to and approved of the manuscript.

## Additional files

The following additional files for this article are available online:

Additional file 1 contains three supplementary figures. Figure S1 shows PC1 from PCA of African Americans based on all genotype data versus African IA from *frappe *analysis. The figure shows near-perfect correlation between PC1 and African IA. Figure S2 shows a *Frappe *analysis of 57 Yoruba, Mandenka, and Bantu speakers, based on estimating admixed ancestry one individual at a time, fixing all others in their defined population. Results show majority assignment to an individual's own population group. Figure S3a shows a PCA of indigenous Africans (n = 94) based on all genotype data. Figure S3b shows a PCA of indigenous Africans (n = 94) based on variable removal of genotype data. Note that the figure shows nearly identical genetic structure to that in Figure 3a, including the separation of Yoruba, Mandenka, and Bantu.

## Supplementary Material

Additional data file 1 Figure S1 shows PC1 from PCA of African Americans based on all genotype data versus African IA from *frappe *analysis. The figure shows near-perfect correlation between PC1 and African IA. Figure S2 shows a *Frappe *analysis of 57 Yoruba, Mandenka, and Bantu speakers, based on estimating admixed ancestry one individual at a time, fixing all others in their defined population. Results show majority assignment to an individual's own population group. Figure S3a shows a PCA of indigenous Africans (n = 94) based on all genotype data. Figure S3b shows a PCA of indigenous Africans (n = 94) based on variable removal of genotype data. Note that the figure shows nearly identical genetic structure to that in Figure 3a, including the separation of Yoruba, Mandenka, and Bantu. Click here for file
