# Supplementary figures and images for: Characterizing the admixed African ancestry of African Americans
Source: Genome Biol. 2009 Dec 22;10(12):R141. doi: 10.1186/gb-2009-10-12-r141 (PMC2812948; doi:10.1186/gb-2009-10-12-r141)

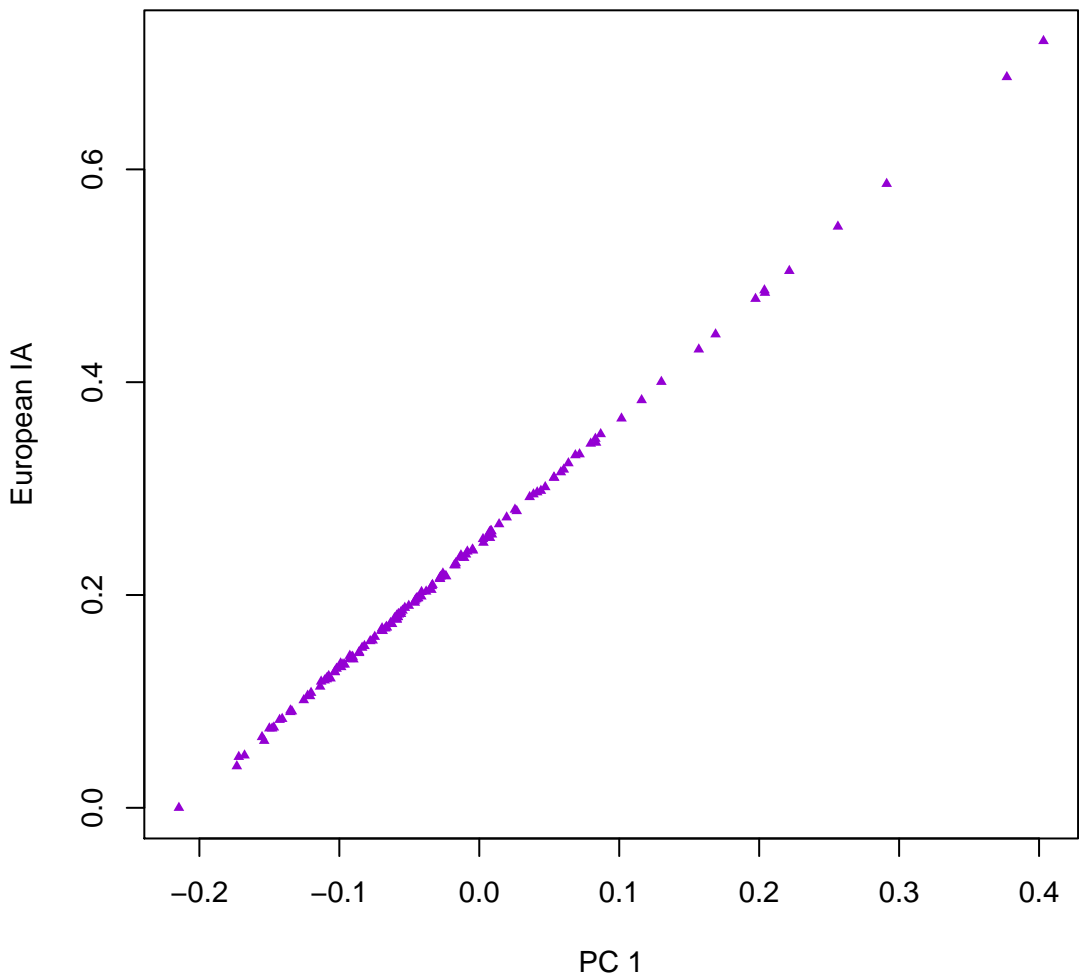

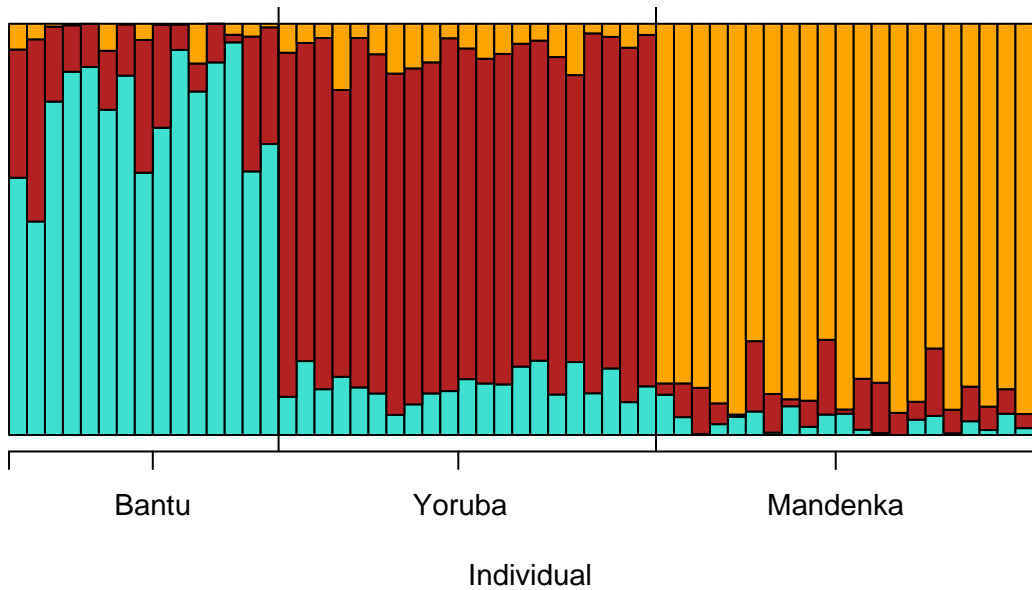

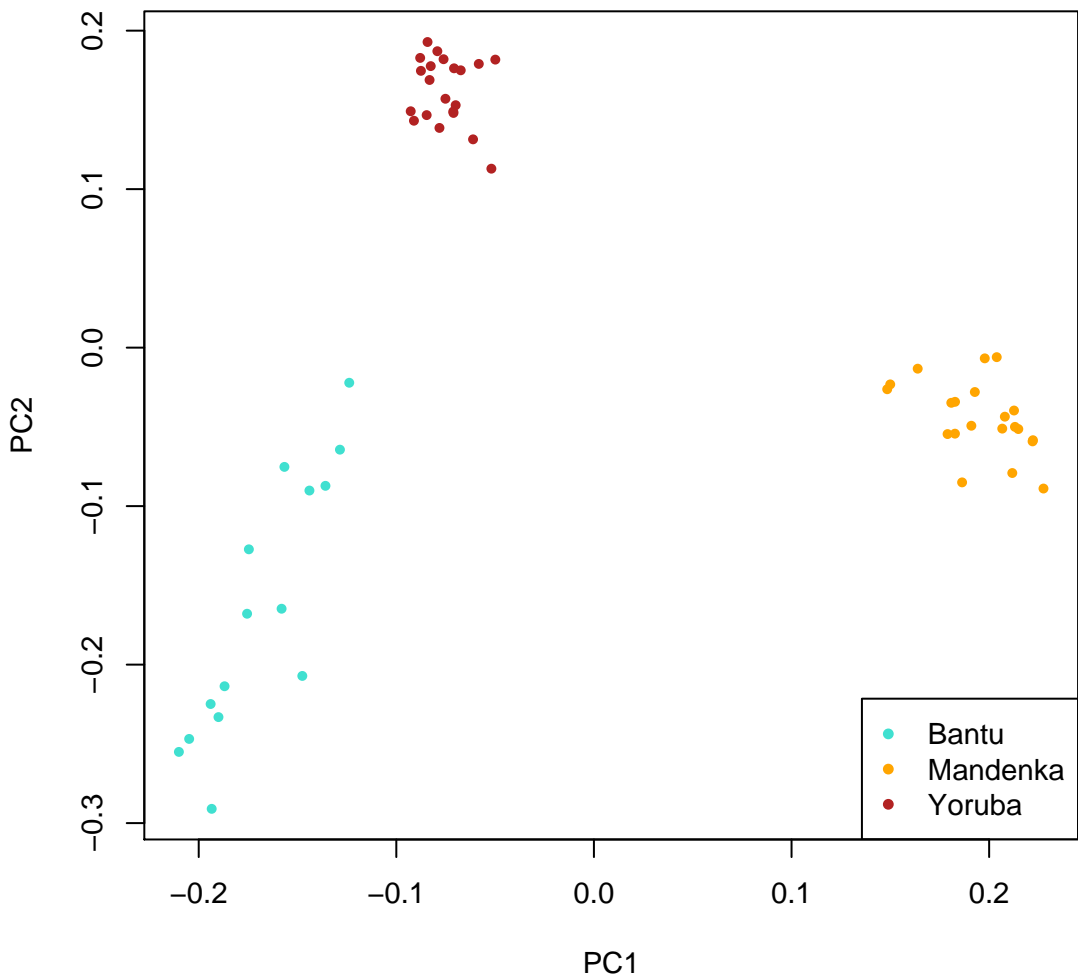

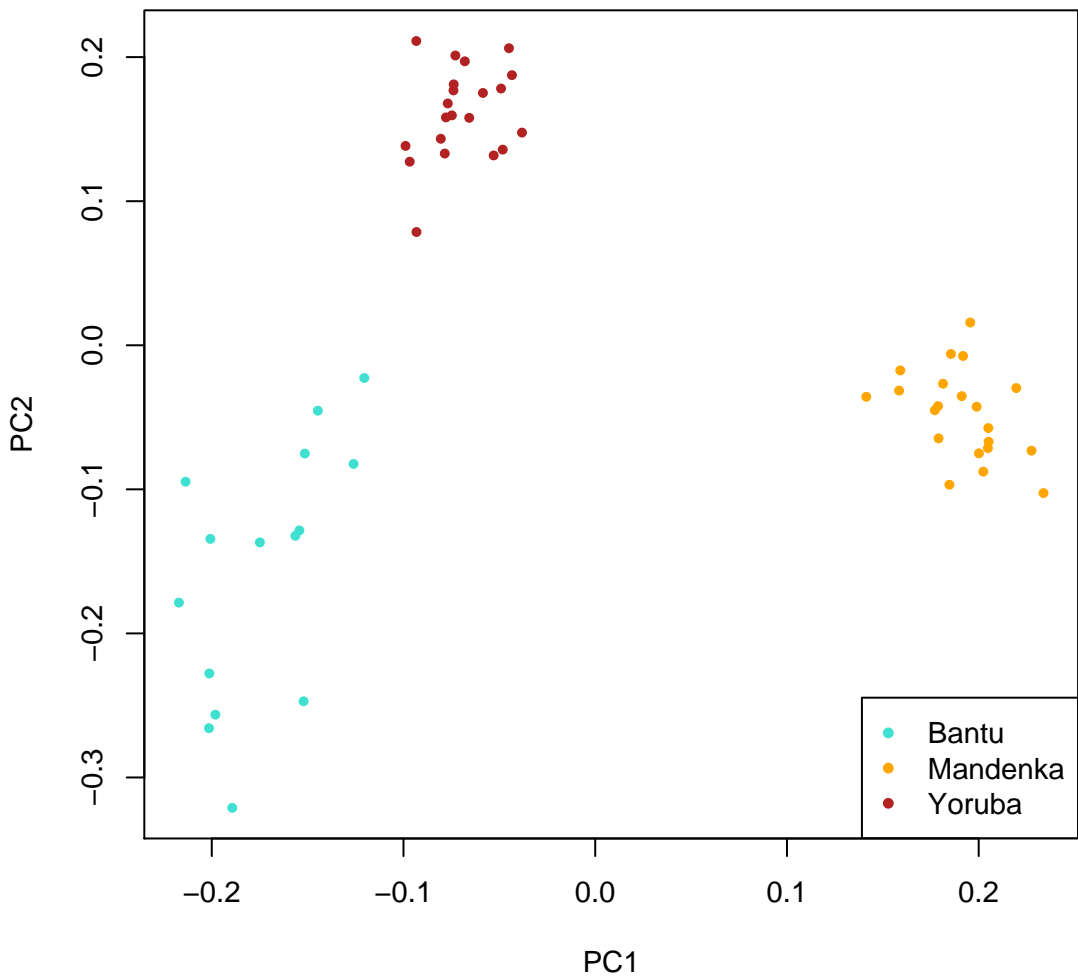

Supplement: Additional data file 1 — Figure S1 shows PC1 from PCA of African Americans based on all genotype data versus African IA from frappe analysis. The figure shows near-perfect correlation between PC1 and African IA. Figure S2 shows a Frappe analysis of 57 Yoruba, Mandenka, and Bantu speakers, based on estimating admixed ancestry one individual at a time, fixing all others in their defined population. Results show majority assignment to an individual's own population group. Figure S3a shows a PCA of indigenous Africans (n = 94) based on all genotype data. Figure S3b shows a PCA of indigenous Africans (n = 94) based on variable removal of genotype data. Note that the figure shows nearly identical genetic structure to that in Figure 3a, including the separation of Yoruba, Mandenka, and Bantu. [file gb-2009-10-12-r141-S1.PDF]
